# Implicit learning across varying temporal scales in individuals with and without mood instability

**DOI:** 10.1016/j.jad.2025.120728

**Published:** 2025-11-17

**Authors:** LZ Atkinson, N Zokaei, P Panchal, N Nelissen, KEA Saunders, MFS Rushworth, D Darby, JR Geddes, CJ Harmer, PJ Harrison, AC Nobre

**Affiliations:** 1 Department of Psychiatry, University of Oxford, Oxford, United Kingdom; 2 Department of Experimental Psychology, University of Oxford, Oxford, United Kingdom; 3 DWP Digital, Quarry House, Quarry Hill, Leeds, United Kingdom; 4 Department of Neuroscience, Monash University, Melbourne, Australia; 5 Department of Psychology and Wu Tsai Institute, Yale University, Connecticut, United States

**Keywords:** Mood instability, remote monitoring, implicit learning, contextual cueing, cognition, attention

## Abstract

Mood instability and cognitive impairment are important features of several psychiatric disorders, including bipolar disorder (BD). However, little is known about how they might be related when measured simultaneously and longitudinally. This 10-week prospective, between-subjects experimental study used digital remote monitoring to capture mood and cognition, and instability in these measures. Two groups of participants were recruited: a ‘high MDQ’ group (n=37), scoring ≥7 on the Mood Disorder Questionnaire (MDQ) with high daily mood instability, and a ‘low MDQ’ group (n=37), scoring ≤5 on the MDQ with low daily mood instability. Five days a week, for 10 weeks, participants completed mood ratings and a contextual cueing task that assessed implicit learning of regularities presented over different temporal scales. Participants with high MDQ scores demonstrated shallower slopes of learning for contextual cueing across days, but not within experimental sessions, compared to individuals with low MDQ scores. Mood instability was not associated with greater instability in reaction times to novel targets. For all participants, learning slopes were significantly steeper for targets repeated more frequently across experimental sessions, e.g. daily compared to alternate days, and within experimental sessions e.g. repeated compared to novel trials, demonstrating successful contextual cueing over varying temporal scales. Our findings highlight that individuals with high levels of mood instability may be less effective at detecting and integrating stimulus regularities across days. Concurrent and high-frequency digital remote monitoring of mood and cognition is feasible and can be used to elucidate relationships between mood instability and cognition.

## 1 Introduction

Mood instability is commonly self-reported in the general population (Marwaha, Parsons, Flanagan, et al., 2013). It is a risk factor for, and a prominent and transdiagnostic feature of, several psychiatric disorders (Broome, Saunders, et al., 2015), notably bipolar disorder (BD) and borderline personality disorder but also schizophrenia, major depressive disorder and anxiety disorders (Gilbert et al., 2005; Marwaha, Parsons, & Broome, 2013; Patel et al., 2015). Mood instability is present in the early emergence of psychiatric disorders (Hafeman et al., 2016; Howes et al., 2011; Miklowitz et al., 2022; Stanislaus et al., 2020), is part of the genetic risk for BD (Ward et al 2020), and is associated with increased risk of relapse and hospitalisation, greater functional impairment and reduced quality of life (Faurholt-Jepsen et al., 2023; Faurholt-Jepsen et al., 2019; Gershon & Eidelman, 2015; O’Donnell et al., 2018; Patel et al., 2015; Perlis et al., 2006; Stange et al., 2016; Strejilevich et al., 2013). The significance of mood instability in psychopathology motivates research to elucidate its associated behavioural and cognitive mechanisms (Harrison et al., 2016).

Impairments in attention, working memory, emotion, and reward processing are known to exist in diagnostic groups experiencing mood instability, particularly mood disorders (Broome, He, et al., 2015). Longitudinal studies in BD, with follow-ups ranging between 1-5 years, have suggested cognitive impairments are stable over time (Samamé et al., 2014, 2022; Szmulewicz et al., 2020). Martino and colleagues found no association between two measures of neurocognitive performance, assessed at least 48 months apart, and a measure of mood instability obtained over the same period (Martino et al., 2018). Nevertheless, assessments repeated months or years apart do not capture variability in cognition that might interact with symptoms fluctuating at a higher frequency (e.g., daily), such as mood instability. It is also possible that mood instability is not associated with global measures of cognitive performance, but instead with more subtle changes in the stability of cognition. Indeed, BD patients demonstrate greater inter-individual variability in measures of visual attention and information processing speed over 2-4 repeated assessments compared to controls (Depp et al., 2008, 2012; Gildengers et al., 2009). Prospective, frequent and simultaneous remote monitoring would help to further understand any relationship between mood instability and cognition.

Attempts to reveal mechanisms that might underlie or maintain mood instability in healthy individuals, rather than as a feature of psychiatric disorders, have focused on disruptions to reward processing (Eldar et al., 2016; Eldar & Niv, 2015; Mason et al., 2017). Eldar and Niv (2015) found that in participants with mood instability, an unexpected win or loss led to greater positive or negative changes in mood and biases in perceived reward value during subsequent learning, a feedback loop that was not observed in individuals reporting stable mood. Greater mood-biased prediction errors might contribute to mood instability, being maladaptive in changing environments. Whilst mood can increase the efficiency of learning about environmental contingencies when the perception of outcomes is appropriate in intensity and duration, greater mood-biased prediction errors might contribute to mood instability (Eldar et al., 2016). Across 50 days of cognitive testing, individuals with high mood instability have been found to display lower risk aversion adaptation than healthy controls (Scholl et al., 2025). In addition, greater levels of mood instability led to less consistent participant choices. However, little research has explored the association between mood instability and cognition in the absence of reward.

Studying mood instability in clinical populations is confounded by many illness and treatment variables. We therefore conducted a longitudinal ten-week study in healthy volunteers (Panchal et al., 2025; Scholl et al., 2025) at low and high risk for BD based on their score on the Mood Disorders Questionnaire (MDQ) (Hirschfeld et al., 2000). The MDQ focuses on past episodes of elevated mood and has a high sensitivity and specificity for BD. Panchal et al., (2025) has previously shown that low and high MDQ scoring groups differ in their mood and mood instability dynamics across ten-weeks.

Here, we used these groups to explore whether the ability to learn and utilize environmental regularities to guide adaptive behaviour, as measured using contextual cueing, is linked to mood instability. Contextual cueing is a phenomenon whereby repeated exposure to consistent contextual information can guide selective attention and facilitate visual-search performance (Chun & Jiang, 1998, 2003; Jiang et al., 2005). In a typical contextual cueing task, participants are faster at locating a target on trials when it appears in the same location within a repeated configuration of distractors, compared to a novel configuration of distractors. When probed, participants are unable to discriminate between repeated and novel configurations presented within experimental sessions, indicating attention is guided by an implicit form of learning (Chun & Jiang, 1998, 2003; Goujon et al., 2015). In a modified version of the contextual cueing task, we manipulated the temporal delay between repetition of regularities. In addition to repeating displays within an experimental session, we also manipulated display repetition across experimental sessions. We were interested in 1) whether individuals showed deficits in utilising regularities presented across different temporal scales (within a task session or across days) to guide attention for adaptive performance; and 2) whether the presence of mood instability led to disruptions in learning and integrating stimulus regularities across days. Specifically, we hypothesised that high MDQ participants might demonstrate slower contextual cueing across days compared to the low MDQ participants, on a similar timescale to changes in mood. In addition, higher levels of mood instability were proposed to be associated with greater variability in participants attentional performance (reaction times) on the task.

## 2 Methods

### 2.1 Participants

Participants, age ≥18, were recruited into 2 groups; a ‘high MDQ’ group, scoring ≥7 on the Mood Disorder Questionnaire (MDQ) and with symptoms co-occurring over the same time period, and a ‘low MDQ’ group, scoring ≤5 on the MDQ. Scores of ≥7 out of 13 on the MDQ have been identified as an optimal cut-off for identifying individuals with symptoms of mood elevation and possible BD (Hirschfeld et al., 2000; Rock et al., 2014; Rock et al., 2010). A cut-off of ≤5 was chosen as presumed low risk for mood elevation and/or BD. Eighty-one participants provided consent to take part and were screened using the Structured Clinical Interview for DSM-IV Axis I Disorders (DSM-IV SCID-I). Participants in the low MDQ group were excluded if they met criteria for a history of, or current DSM-IV axis I psychiatric disorder or had a first degree relative with BD. Participants in the high MDQ group were excluded if they met criteria for a history of, or current DSM-IV axis I psychiatric disorder other than BDI or II, MDD or anxiety disorder. High MDQ participants remained eligible if symptomatic at the time of baseline screening, unless they were deemed to require psychiatric treatment. See Panchal et al. (2025), Scholl et al. (2025) and Supplementary Materials for detailed exclusion criteria. Two participants were excluded during screening (1 high and 1 low MDQ) and one withdrew consent for data use (1 high MDQ). Additionally, four high MDQ participants were excluded from the current analysis for providing mood and cognitive task data for less than three out of ten weeks. Complete data were available from 74 participants (37 high and 37 low MDQ). Study approval was granted by Oxfordshire Research Ethics Committee (MSD-IDREC-C2-2014-023).

### 2.2 Study design

At an in-person baseline visit participants provided demographic information, underwent structured clinical interviews, completed self-report questionnaires including the Affect Intensity Measure (AIM) and the short-form Affect Lability Scale (ALS-SF), and completed a visual attention task to assess various parameters of sensory processing and attentional performance at baseline (section 2.2.1.). Participants were provided with an iPad and received training to complete at-home data collection on a web interface specifically designed for the study. For 10 weeks following the baseline visit, participants completed short mood ratings and cognitive tasks 5 days a week.

Daily mood was assessed using the 10-item International Positive and Negative Affect Scale (I-PANAS-SF) (Thompson, 2007). The 5-positive and 5-negative affect items appeared in a pre-determined order each day: upset, hostile, alert, ashamed, inspired, nervous, determined, attentive, afraid and active. Each item was rated on a 5-point Likert scale (1 = Very slightly/Not at all; 5 = Extremely). Four novel app-based tasks were developed to assess cognition, including the contextual cueing task (section 2.2.2). Each task took 1-2 minutes to complete each day. Mood ratings were completed prior to cognitive tasks. If participants missed too many days in a row e.g. > 2 days, they were prompted to increase compliance. All data were automatically recorded and stored.

#### 2.2.1 Visual attention task

A visual attention task, based on the Theory of Visual Attention (TVA) (Bundesen, 1990), was used at baseline to quantify sensory and attentional parameters including visual short-term memory (VSTM) capacity, speed of visual processing, threshold of conscious perception of objects, and efficiency of top-down attentional control. The TVA is based on accuracy, whereby attentional parameters are unconfounded by motor function (e.g. reaction time (RT)). The Combi TVA (Vangkilde et al., 2011) includes two experimental conditions; whole-report trials, in which all stimuli are targets, and partial-report trials, which include targets and distractors. Typically, targets are letters and distractors are digits, as utilised in this task.

**Figure 1:**
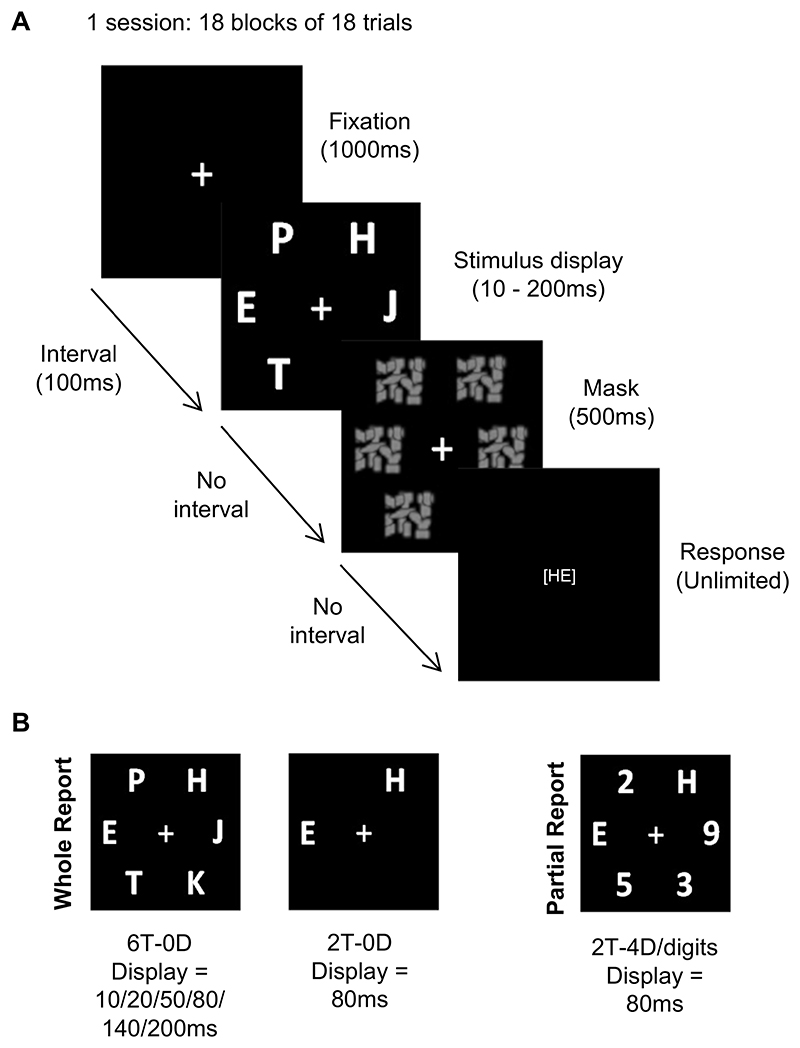
TVA task. **A**. Sequence through one single whole report trial of the task. **B**. Possible whole and partial report trial types, and their stimulus exposure durations.

Participants were required to identify and report any letters that occurred across three conditions: 1) 6T-0D: whole-report trials of six targets presented at varying durations of 10, 20, 50, 80, 140, and 200 ms in the absence of any distractors; 2) 2T-0D: whole-report trials of two targets presented at 80 ms in the absence of any distractors; 3) 2T-4D: partial-report trials of two targets, presented at 80 ms, in the presence of 4 digit distractors. Participants completed 18 blocks of 18 trials, a total of 324 trials of which 162 were whole-report six-target trials, 81 were whole-report two-target trials and 81 were partial-report digit distracter trials (Figure 1). For further task stimuli details see the Supplementary Material methods.

Both whole- and partial-report trials were randomised within each block. At the end of each block, participants were given feedback on their accuracy rate to report correct target letters. In order to analyse the TVA, it is desirable to equate the criterion used across participants. Participants were asked to maintain an accuracy range of 80-90% in their reporting of targets; if their accuracy was higher, they were encouraged to be less conservative and guess more; if their accuracy was lower, they were encouraged to guess less. Participants were informed that their RT was not recorded. The task took approximately 40 minutes.

#### 2.2.2 Contextual cueing task

**Figure 2:**
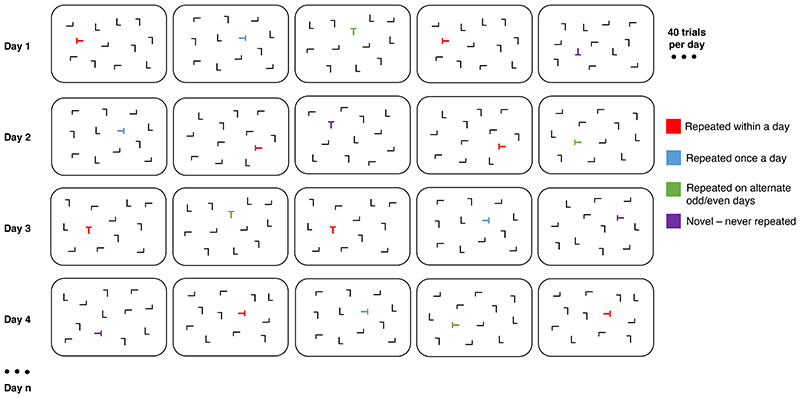
Contextual cueing task. Task conditions, varying over trials and across days. Each trial display contained 12 stimuli (1 target ‘T’ and 11 ‘L’ shape distractors). Red targets (‘T’) portray ‘within-day’ trials (only two trials per day illustrated). One configuration of identical target and distractor locations repeated in ten trials within a day (testing session) but did not appear on any other day. Blue targets portray ‘once daily’ trials (only one trial per day illustrated). Ten distinct configurations were repeated once per day, every day. Green targets portray ‘alternate day’ trials (only one trial per day illustrated). Ten distinct configurations repeated on odd days (shown on day 1 and 3) and ten distinct configurations repeated on even days (shown on day 2 and 4). Purple targets portray ‘novel’ trials (only one trial per day illustrated). Ten distinct configurations were presented each day and never repeated.

The contextual cueing task (Chun & Jiang, 1998) was adapted to include array configurations that repeated across days, in addition to within testing sessions. This allowed for the investigation of potential disruption to learning and utilizing contextual regularities over different temporal scales to guide attention. In the gamified app, the contextual cueing task was called “Whack-a-T”. Participants were required to identify and tap the location of one target (a ‘T’ presented in any orientation), hidden among an array of 11 distractor stimuli (‘L’s presented in any orientation) in four different conditions: 1) novel - 10 distinct configurations presented each day that were never repeated; 2) alternate day - 10 distinct configurations repeated on odd days & 10 distinct configurations repeated on even days; 3) daily – 10 distinct configurations repeated once per day, every day; 4) within-day – 10 repetitions of 1 configuration within a day (testing session) that never repeated on another day. A total of 40 trials were presented each session across the four conditions (Figure 2).

For the three conditions that repeated over different temporal scales (within-day, daily, and alternate days), a target always appeared in the same location within a given configuration. The identities and locations of the distractors were also preserved across repetitions. The order of trials was randomised with one constraint - within-day trials never appeared consecutively to circumvent participants noticing within-session trial repetitions. For further task stimuli details see the Supplementary Material methods.

Participants were instructed to ‘tap on the T shape among the L shapes’. There was a prestimulus interval of 500 ms before the first trial. The background of the search array was black, and stimuli were white. When participants correctly selected a target, it turned yellow; if they incorrectly selected a distractor, it turned red. After an inter-trial interval (ITI) of 1000 ms, the screen advanced to the next trial. After 40 trials, the task ended. RTs to finding and tapping a target or distractor were recorded as the primary outcome. Accuracy of selecting the target over a distractor was recorded as a secondary outcome.

### 2.3 Statistical analyses

#### 2.3.1 Mood data

Only mood data accompanied by cognitive task data on the same day was included in the current analyses. Overall instability and mean of daily positive and negative affect scores on the I-PANAS-SF were calculated. Instability was quantified using a time-adjusted root mean square of successive difference (tRMSSD) (Taquet et al., 2023):

$$tRMSSD=\sqrt{\frac{1}{N}\sum_{i=1}^{N-1}{\left(\frac{x_{i+1}-x_{i}}{t_{i+1}-t_{i}}\right)}^{2}}$$

where *x_i_* (*i=1*,…, *N*) indicates the *N* mood ratings and *t*_i_ (*i=1*,…,*N*) indicates the timestamps at which the mood ratings were submitted, such that *t*_i+1_–*t*_i_ is the difference in time between two subsequent datapoints. The tRMSSD is obtained by calculating each successive difference in mood ratings over each successive time difference between ratings. This value is subsequently squared, and the result is averaged before the square root of the total is calculated. Incorporating time into the calculation of RMSSD minimises artefactual increases in cases of missing data between mood ratings. For additional analyses of week-by-week mood data see Panchal et al., 2025.

#### 2.3.2 Visual attention task data

Analysis of variance (ANOVA) was used to analyse accuracy data (percentage of correct letters reported). For six-target whole report trials, exposure duration was included as the within-subject factor and for two-target partial report trials, distractor type was included as the within-subject factor. In both models, group was included as a between-subject factor.

A TVA model (Bundesen, 1990), based on a maximum-likelihood fitting procedure, was used to derive four parameter estimates: (1) *K*, the capacity of VSTM measured by the number of letters reported from all targets; (2) *C*, the speed of visual processing measured in letters processed per second; (3) *t0*, the stimulus duration threshold for conscious perception; and (4) α, the efficiency of top-down selectivity (or the distractibility index) estimated by comparing performance in partial report trials (two targets and four distractors) with performance in whole report trials (two targets no distractors). For further information see (Bundesen & Habekost, 2014; Dyrholm et al., 2011; Kyllingsbæk, 2006; Vangkilde et al., 2011).

Data extraction and fitting procedures were performed using Matlab (version 2016b) and the LibTVA package (Dyrholm et al., 2011; Kyllingsbæk, 2006). TVA models were fit for partial report distractors (digits), with the model including the whole report data. Basic model parameters *K, C, t0* and α were compared between groups.

#### 2.3.3 Contextual cueing task

Data cleaning and slope fitting procedures were carried out in Matlab (version 2021b). Statistical tests and plotting were conducted in *R* (version 4.1.2; R Core Team, 2019).

Task data were cleaned by removing excessively short or long RTs, <0 ms and >8,000 ms (167 trials, 0.12%). On 336 trials (0.24%), a technical error meant that correct or incorrect responses were recorded as NaN values which were removed from the data. For analyses of RT data, error trials were further excluded (474 trials across all participants, 0.35%). For each participant, RTs >3 SDs from the mean RT of each condition were removed (2378 trials across all participants, 1.73%). Overall, 2.44% of the data were removed during cleaning.

For each participant, RT data were chunked into 10 weeks based on how many task sessions were completed during a 7-day period. Within each epoch, the mean RT was calculated for the novel, alternate-day, and daily conditions. To assess change in RT as a function of learning, learning-slope parameters were estimated by applying the power-law function to RTs of novel, alternate-day, and daily conditions. Quantifying learning in this way has previously been demonstrated in the contextual cueing literature (Chun & Jiang, 2003). Also observed by Chun and Jiang (2003), the power law function yielded better fits to the current data than exponential or linear functions, demonstrated by lower mean squared error in each condition. For each participant, search slopes were derived from the best-fit line of mean RTs across all days, for each condition. Data were normalised by subtracting the slope of the alternate day and daily conditions from the novel condition for each participant.

For within-day trials, learning-slope parameters were estimated by applying the power-law function to derive search slopes from the RTs of each trial (1-10) for novel and within-day conditions each day. Data were normalised by subtracting the slope of the within-day condition from the novel condition for each participant.

Repeated-measures ANOVA, using the afex package (version 1.0-1, Singmann et al., 2021), was used to investigate accuracy (percentage of ‘T’ targets identified), mean RT for novel trials, and slopes of learning across days, with cueing condition or week included as a within-subject factor and group as a between-subject factor. Where assumptions of sphericity were violated, Greenhouse-Geisser adjustments were applied. Significant interactions were investigated with pairwise tests using the emmeans package (version 1.7.2; Lenth, 2022) with Tukey post-hoc correction. The ggplot2 package (version 3.3.5; Wickham, 2016) was used to plot results. Independent samples t-tests were used to compare differences in the mean normalised slope within day between groups. Following tests of normality, Pearson/Spearman correlations were performed as appropriate to assess the presence of any relationship between task performance and mood instability in positive and negative affect.

## 3 Results

Demographic characteristics of participants (37 high and 37 low MDQ) are presented in Table 1.

### 3.1 Mood and cognitive task compliance

There were no differences between high and low MDQ participants in the number of mood ratings and cognitive tasks completed each week (*F*(1,65)=.36, *p*=.55, ηp^2^=<.01), or group-by-week interaction (*F*(6.32, 410.95)=.95, *p*=.47, ηp^2^=.01). However, compliance was significantly different across weeks in the study (*F*(6.32,410.95)=3.55, *p*=.002, ηp^2^=.05). Compared to week 1, compliance was significantly lower in weeks 2 (M=4.15, SE=.19, *p*=.01) and 10 (M=4.22 SE=.14, *p*=.002), see Supplementary Figure 1.

### 3.2 Instability, variability and mean of PANAS-SF ratings

**Figure 3:**
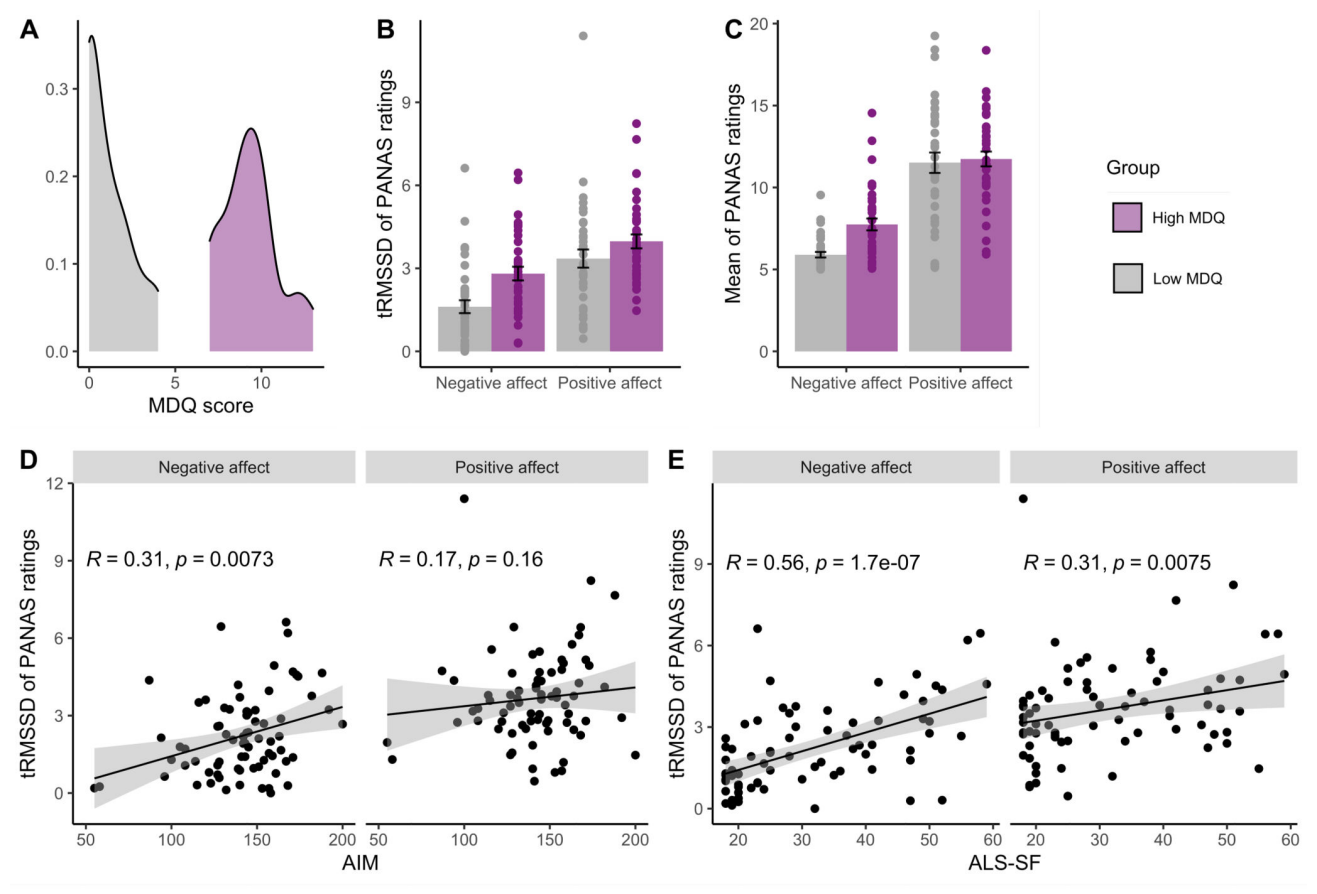
Mood questionnaire outcomes. **A**. Density of Mood Disorder Questionnaire (MDQ) scores in low and high MDQ groups. **B**. Group mean instability of negative and positive scores on the Positive and Negative Affect Scale, Short Form (PANAS-SF). Individual participant instability quantified using time adjusted Root Mean Square of Successive Differences (tRMSSD) of mood scores over ten weeks. **C**. Group mean of negative and positive PANAS-SF scores over ten weeks. Error bars on figures B-C represent ±SE of mean. **D**. Whole sample Spearman correlation between baseline scores on the Affect Intensity Measure (AIM) and instability of negative and positive PANAS-SF scores. **E**. Whole sample Spearman correlation between baseline scores on the Affect Lability Scale-Short Form (ALS-SF) and instability of negative and positive PANAS-SF scores.

The high MDQ group demonstrated greater overall instability, as measured by tRMSSD, in negative affect compared to the low MDQ group, (2.81 ± 1.51 vs 1.61 ± 1.42, *t*(72)=-3.52, *p*<.001, *d*=.82) but not greater overall instability in positive affect (3.97 ± 1.53 vs 3.35 ± 1.99, *t*(72)=-1.5, *p*=.14, *d*=.35, Figure 3B). Further, the high MDQ group experienced greater mean negative affect scores (7.75 ± 2.21) than the low MDQ group (5.90 ± 1.03) (*t*(72)=-4.62, *p*=<.001, *d*=1.07). However, high and low MDQ groups did not differ in mean positive affect scores (11.74 ± 2.75 vs 11.51 ± 3.79 respectively, *t*(72)=-.30, *p*=.77, *d*=.07, Figure 3C).

There was a significant positive correlation between higher MDQ scores and increased levels of negative mood instability (*r_s_*(74)=.39, *p*=.001). A positive correlation between MDQ scores and positive mood instability was observed, but did not reach statistical significance (*r_s_*(74)=.22, *p*=.06). Baseline scores of affect intensity, as measured by the AIM, were significantly positively correlated with instability in negative affect (*r_s_*(74)=.31, *p*=.007) but not instability in positive affect (*r_s_*(74)=.17, *p*=.16, Figure 3D) measured across the study. Baseline scores of affect lability, as measured by the ALS-SF, were significantly positively correlated with instability in both negative (*r_s_*(74)=.56, *p*<.001), and positive affect (*r_s_*(74)=.31, *p*=.008, Figure 3E). As expected, there was a significant positive relationship between total AIM scores and total scores on the ALS-SF (*r_s_*(74)=.42, *p*<.001).

### 3.3 Contextual cueing task results

#### 3.3.1 Accuracy

Overall accuracy for detecting targets in visual search across conditions was high, showing accuracy of ~95% (see Supplementary Figure 2). There were no differences in percentage accuracy in locating the target between high and low MDQ groups (*F*(1,72)=.04, *p*=.85, ηp^2^=<.01) or cueing condition (*F*(2.41,173.30)=1.15, *p*=.32, ηp^2^=.02), nor was there a group by condition interaction (*F*(2.41,173.30)=0.69, *p*=.53, ηp^2^=<.01). The subsequent analyses of RT data include only trials in which the target ‘T’ was correctly identified.

#### 3.3.2 RTs: Novel trials – overall attention performance

The novel condition of the contextual cueing task provided a baseline measure of attentional performance, in the absence of implicit learning. Mean RTs of novel trials across the 10 weeks were compared between groups. There were no differences in RTs between low and high MDQ groups (*F*(1,62)=.35, *p*=.56, ηp^2^=<.01), suggesting comparable visual search performance over the study. A significant effect of week revealed that participants became significantly faster at identifying the target ‘T’ over time, an expected practice effect on the task (*F*(5.37, 333.20)=5.15, *p*<.001, ηp^2^=.08). Post-hoc comparisons revealed that RTs in week 1 (M=1362, SE=27.0) were significantly slower than all other weeks, except from week 8 (M=1287, SE=32.7). There was no significant group-by-week interaction (*F*(5.37,333.20)=.61, *p*=.71, ηp^2^=<.01), see Supplementary Figure 3.

#### 3.3.3 RTs: Daily, alternate-day, and novel trials – contextual cueing across days

**Figure 4:**
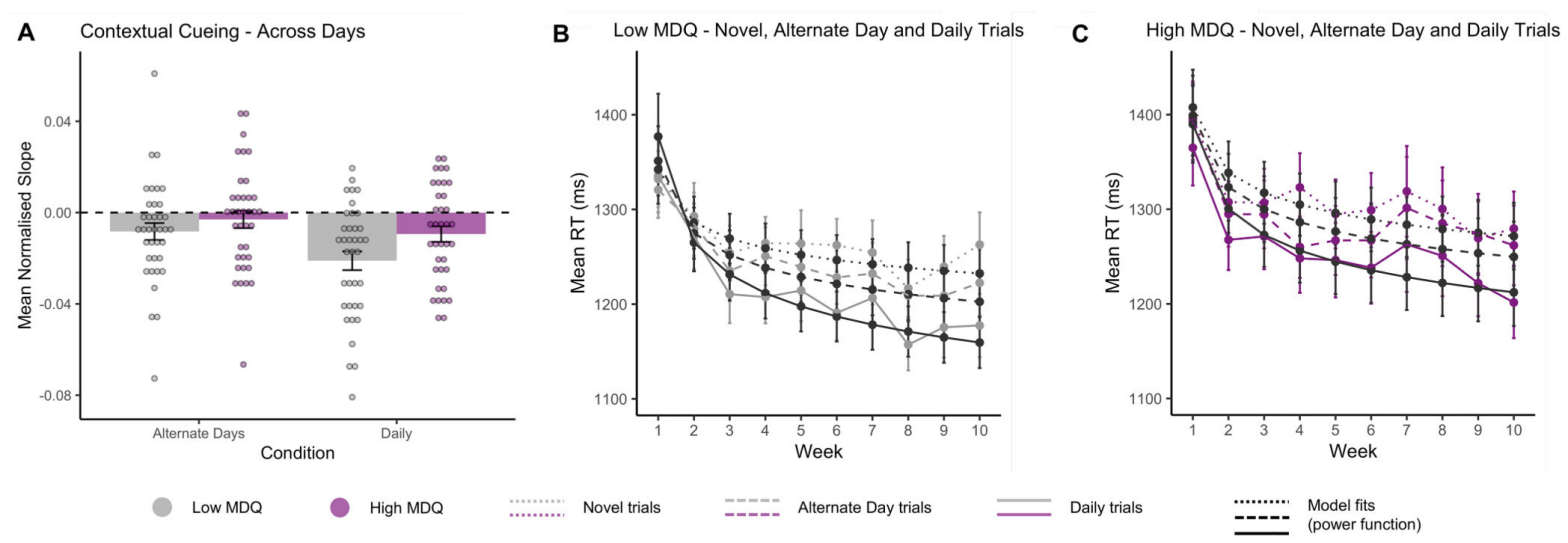
Contextual cueing across days. **A**. The contextual cueing benefit for identifying targets in repeated configurations (daily and alternate days) relative to novel configurations. The power function was independently fitted to daily, alternate day and novel trials for each participant. The resulting slope, representing the rate of learning, was subsequently normalised by subtracting the slope of daily and alternate day trials from the mean slope of novel trials for each participant. More negative values represent a faster rate of learning. Data is shown for both low (grey) and high (purple) MDQ groups. **B**. Mean RT in milliseconds (ms) of novel, alternate day, and daily repeated trials by week for the low MDQ group. Actual RT data (grey lines) and model fits (black lines) for novel (dotted), alternate day (dashed) and daily (solid) trial types. **C**. Same as B. for the high MDQ group (purple lines). Error bars represent ±SE of mean.

The core analysis of interest investigated whether contextual cueing was possible across different timescales and, specifically, whether there were differences between low and high MDQ groups in implicit learning of regularities across days. The following analyses therefore compared slopes of learning in the daily, alternate-day, and novel conditions of the task. To observe benefits in RTs facilitated by contextual cueing, data were normalised by subtracting the mean of the slope of the novel condition from the daily and alternate day conditions for each participant. The high MDQ group demonstrated shallower slopes of learning (Mean=-.006, SE=.003) compared to the low MDQ group (Mean=-.015, SE=.003), (*F*(1,72)=3.72, *p*=.05, ηp^2^=.05 (see Figure 4A). In addition, slopes for the normalised daily condition (Mean=-.015, SE=.003) were steeper than those of the normalised alternate day condition (Mean=-.006, SE=.003), (*F*(1,72)=10.24, *p*=.002, ηp^2^=.12); cueing conditions repeated more often (daily<alternative days) lead to facilitated recognition of targets, demonstrating contextual cueing across days. There was no interaction effect between group and condition (*F*(1,72)=1.14, *p*=.29, ηp^2^=.02). Mean RTs across weeks, and fitted slopes of learning for each group and condition are shown in Figure 4B-C.

#### 3.3.4 RTs: Within day and novel trials – contextual cueing within a session

**Figure 5:**
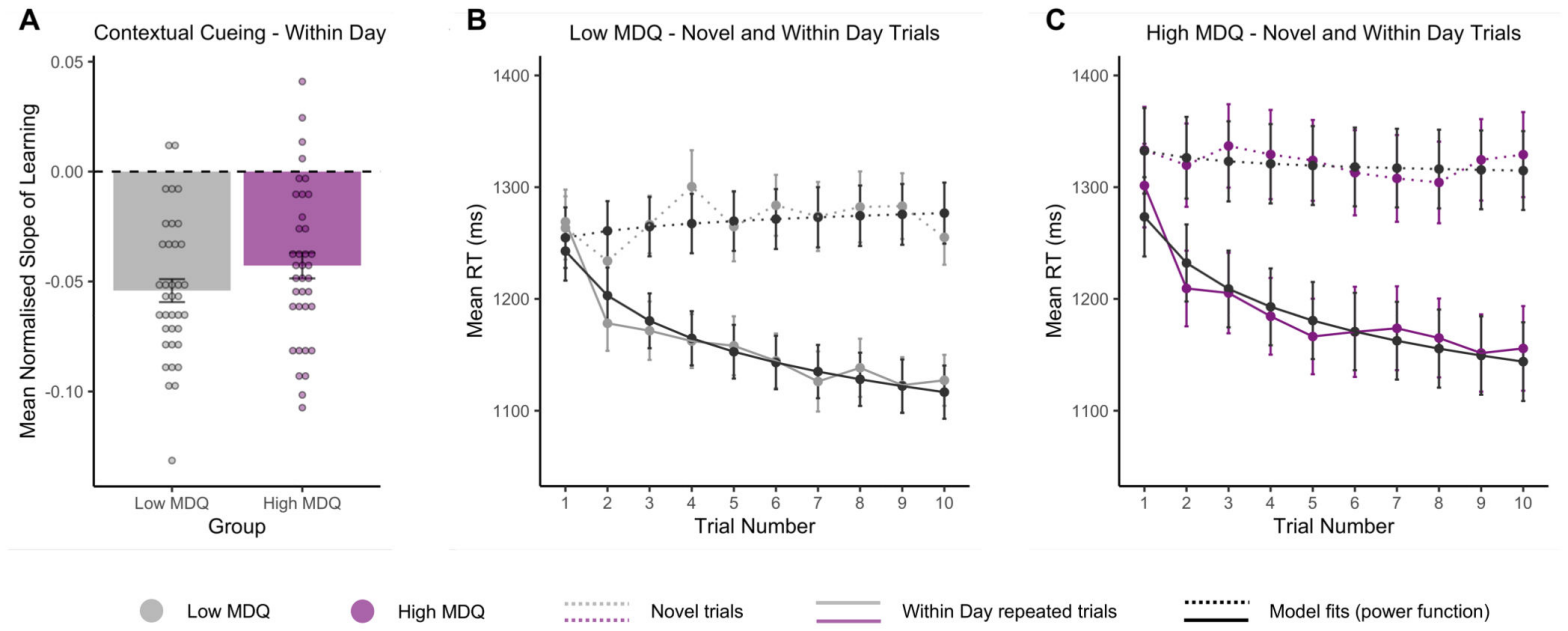
Contextual cueing within a testing session. **A**. The contextual cueing benefit for identifying targets in within-day repeated configurations relative to novel configurations. The power function was independently fitted to within-day trials and novel trials for each participant. The resulting slope, representing the rate of learning, was subsequently normalised by subtracting the mean of the slope of within day trials from the slope of novel trials for each participant. More negative values represent a faster rate of learning. Data is shown for both low (grey) and high (purple) MDQ groups. **B**. Mean RT in milliseconds (ms) of novel and within day repeated trials across all sessions for the low MDQ group. Actual RT data (grey lines) and model fits (black lines) for novel (dotted) and within day (solid) trial types. **C**. Same as B. for the high MDQ group (purple lines). Error bars represent ±SE of mean.

The following analyses investigated the presence of the typical contextual cueing effect within a testing session; faster identification of targets in repeated configurations compared to novel configurations. Specifically, it was of interest whether this effect was different between high and low MDQ groups. The following analyses therefore compared slopes of learning in within-day and novel conditions of the task. As in the previous analysis, data were normalised by subtracting the mean of the slope of the novel condition from the within day condition for each participant. There were no group differences in learning slopes between high and low MDQ groups (*t*(72)=-1.46, *p*=.15, *d*=.34) (Figure 5A) although slopes were slightly steeper for individuals with low MDQ (M=-.054, SE=.005) than high MDQ (M=-.043, SE=.006). Mean RTs across trials, and fitted slopes of learning for each group and condition are shown in Figure 5B-C.

### 3.4 RTs: instability of novel trials

For each participant, instability in mean RTs to novel trials each day (in the absence of learning), was quantified using tRMSSD. One high MDQ participant was excluded due to showing extreme outlier values in instability of mean RT for novel trials. No group differences were observed (*t*(71)=-.57, *p*=.57, *d*=.13).

### 3.5 Relationship between cognition and mood instability

No significant associations were observed between instability in mean RT for novel trials each day and negative mood instability (*r_s_*(71)=-.10, *p*=.39) or positive mood instability (*r_s_*(71)=-.16, *p*=.18) (see Supplementary Figure 4). Further, there were no significant associations between instability in the within-day cueing benefit and negative mood instability (*r*(72)=0.2, *p*=.08) or positive mood instability (*r*(72)=.06, *p*=.61) (see Supplementary Figure 5).

### 3.6 Visual attention task results

Due to an error during data collection, CombiTVA data were only available from 65 participants (35 high MDQ; 30 low MDQ). No differences in baseline demographics or mood data were observed in this subsample compared to the full sample (see Supplementary Table 1).

There were no significant group differences in mean accuracy of reported targets for whole report (*F*(1,63)=.41, *p*=.52, ηp^2^=<.01) or partial report trials (*F*(1, 63)=.34, *p*=.56, ηp^2^=<.01) (see Supplementary Figure 7A-B). Further, model fitting of TVA parameters found no significant differences between high and low MDQ groups in visual short-term memory capacity (VSTM, *K)* (*t*(63)=.19, *p*=.85, *d*=.05), mean visual processing speed (*C*) (*W=494, p*=.84, r=.03), or the distractibility index (α) (*W*=585.5, *p*=.43, r=.10). There was a marginally significant difference in perceptual threshold (*t*0), with low scoring MDQ participants displaying lower minimum exposure duration to identify targets compared to high scoring MDQ participants (*W*=371, *p*=.06, r=.23) (See Supplementary Figure 7C-F and Supplementary Table 2).

## 4 Discussion

This study investigated whether mood instability compromised cognitive performance on a visual-search task within individual sessions, or impacted the ability to learn and integrate contextual regularities across days to guide attention benefits. In a novel adaptation of the contextual-cueing task, we manipulated how often target locations were repeated within specific distracter configurations, enabling learning over short (within-day) and long (across-day; daily and alternate days) intervals. To assess the degree of implicit learning, we compared the time taken to identify targets in repeated contexts, to novel contexts free of any learning. Our results showed that participants with high MDQ scores, experiencing greater levels of mood instability, demonstrated shallower slopes of learning for contextual cueing across days, but not within experimental sessions, compared to those with low MDQ scores.

The core finding from this study suggests that individuals with high mood instability may be less effective at detecting and integrating stimulus regularities implicitly learnt across days. Impairments in learning regularities over longer temporal intervals could also affect mechanisms underlying day-to-day mood changes by slowing the learning and integration of information that supports mood regulation. Moreover, heightened mood instability may further disrupt learning by altering context, thereby reducing context-appropriate transfer of learning (Godden & Baddeley, 1975; Smith & Vela, 2001).

Across-day contextual learning deficits may reflect abnormalities in the consolidation, maintenance, or linkage of context-target associations. Retention of implicitly learned spatial regularities depends on hippocampal-neocortical consolidation processes that occur during offline periods, including sleep. Given that sleep disturbances are more common in individuals experiencing mood instability than those not (McDonald et al., 2017), and that alterations in hippocampal structure and connectivity have been reported in mood disorders (Chen et al., 2018), consolidation processes may be compromised by reducing the strength and reactivation of relevant memory representations in individuals with high mood instability. Additionally, faster decay of memory traces or greater susceptibility to interference from internally focused or mood-related thought patterns could compete with task-relevant reactivation, further weakening consolidation and transfer of learning across sessions.

Such disruptions may have important clinical implications. Adaptive emotional and behavioural regulation strategies rely on gradually learning which contexts or actions promote mood stability or improvement. If individuals with high mood instability learn contextual regularities more slowly or inconsistently, they may find it more difficult to develop or maintain effective regulation strategies, potentially contributing to day-to-day fluctuations in mood. Such difficulties may also reduce the effectiveness of psychological interventions, such as cognitive-behavioural therapy, which rely on recognising consistent associations between contexts, behaviours and emotional outcomes across sessions. Approaches that target consolidation or context integration such as cognitive training, sleep optimisation, or neuromodulation, may therefore help strengthen contextual learning across days and, in turn, support improved mood stability.

High and low MDQ groups showed similar rates of contextual cueing acquisition within a day. This contrasts with prior research reporting severely impaired contextual cueing within experimental sessions in major depressive disorder, and slower acquisition of contextual cueing within experimental sessions in schizophrenia (Lamy et al., 2008). Several factors may account for this discrepancy. Our sample comprised largely of subclinical or at-risk individuals. These individuals are likely to show less pronounced deficits than confirmed clinical populations. In addition, among the nine participants (out of 37) who met criteria for a psychiatric disorder, diagnoses were heterogeneous. Subtle differences in contextual cueing impairments shown to be important between psychiatric populations (Lamy et al., 2008) may therefore have been obscured. Finally, methodological differences such as fewer trials per condition, shorter experimental sessions, and the variability inherent in unsupervised remote testing may have reduced our ability to detect small impairments in within-day contextual cueing, compared with controlled single-session lab-based contextual cueing tasks using larger trial counts.

Previous work exploring the longitudinal relationship between mood and cognition in clinical populations have used long intervals between assessments (e.g. months/years) (Budde & Schulze, 2014; Chaves et al., 2011; Martino et al., 2018; Samamé et al., 2014; Santos et al., 2014), or used few repeated measures (Depp et al., 2012), limiting sensitivity to change or instability in cognitive performance over time. In contrast, we demonstrated the feasibility of frequent remote monitoring of mood and cognitive performance over ten weeks (Panchal et al., 2025; Scholl et al., 2025) - the longest known period of simultaneous monitoring of mood and cognition. Compliance to monitoring was high, with 92% in the high MDQ group and 94% in the low MDQ group, comparable to other longitudinal smartphone-based daily mood reporting studies (e.g. 12 weeks, Duffy et al., 2019; Tsanas et al., 2016).

At baseline, we observed no differences between groups in perceptual threshold, speed of visual processing, top-down selectivity, or working memory capacity as measured by the combiTVA task. A limitation of this study is that we did not repeat this task or include other direct assessments of attention over the course of the 10-weeks to observe potential changes in attentional capacity over time. However, we did investigate group differences in RTs to identifying targets within novel configurations of the contextual cueing task, which provided an indirect measure of attentional performance in the absence of implicit learning. We found no group differences in RTs to novel trials across weeks. This indicates comparable visual search performance and provides evidence to suggest our results are specific to implicit learning over time, rather than general attentional processing parameters impacting performance within a given session.

As instability in RTs for novel trials did not differ between high and low MDQ participants and was not associated with mood instability in either positive or negative affect, we conclude that our high MDQ sample do not exhibit a general deficit in the stability of cognitive performance. A previous study reported high within-participant correlations on attention tasks such as the Trail Making Test repeated 6 times over 15-20 years in patients with BD (Burdick et al., 2006). Conversely, Depp et al., (2012) found greater inter-individual variability in cognitive performance across 2-4 sessions over six months in individuals with euthymic BD, compared to healthy controls. However, their performance measure was a composite score across tasks such as Digit Symbol-Coding, Trail Making Test, verbal fluency, verbal learning, response inhibition and task switching, making it unclear whether instability in cognition was driven by specific, or generalised, domains. Consistent with our findings, they did not find an association between changes in cognitive performance and changes in mood symptoms over time (Depp et al., 2012). Our study addresses limitations in previous research by using more frequent assessments and incorporating time-sensitive methods to quantify instability.

We provide novel evidence that contextual cueing can occur across days. Previous studies have varied the frequency and ratio of repeated configurations within experimental sessions to influence the degree of learning-based attention (Zang et al., 2018; Zinchenko et al., 2018). For example, Tseng et al., (2011) presented novel configurations alongside four configurations that repeated three times, four that repeated twice and four that repeated once per block. They observed more efficient search for configurations presented more frequently, attributing this to stronger associative memory (Tseng et al., 2011). In the present study, a similar pattern was observed across days, rather than task blocks, with faster search times for targets within configurations presented daily compared to on alternate days.

The within-day contextual cueing effect in the present task was robust, consistent with prior studies that detect it after relatively few trials (Chun & Jiang, 1998; Tseng & Li, 2004). Ten trials were presented for repeated and novel conditions each day, ensuring testing sessions were brief and engaging to complete at-home. Further, RTs were comparable to those in lab-based contextual cueing experiments. Whilst contextual cueing provides a measure of implicit learning thought to be less susceptible to ceiling effects (Klinge et al., 2018), we did not directly test whether participants had explicit awareness of repeated configurations. Previous research has shown that contextual cueing information is consciously retrievable as the number of trials is increased (Smyth & Shanks, 2008). Although we used only a small number of trials per condition (10) each day, participants did repeat some trials for up to 50 sessions. Thus, we are limited in our certainty that contextual cueing was fully implicit, especially given the repeated exposure of trials across days.

Our findings are specific to implicit learning across time in the absence of feedback or reward. Nevertheless, in healthy individuals without mood disturbances, rewarding contexts can accelerate implicit learning within experimental sessions, and consistent or inconsistent feedback can result in faster and slower learning, respectively (Tseng & Lleras, 2013). Our work complements and extends observations from the computational psychiatry literature that highlight the close and dynamic interplay between cognition and mood. The deficits we observed in benefiting from spatial configural regularities to guide attention during visual search may tap into some similar processes found to be disrupted in probabilistic reward-learning tasks. Mood-related dysregulation could compromise various computational parameters linked to improving performance based on prior experience (Christie, 2021). For example, fluctuations in mood could change the weighting of information across time and destabilise learning (Eldar et al., 2018). Individuals may overestimate the volatility in the arrays, leading to poorer contextual learning (Vinckier et al., 2018). Of note, our study extends the investigation of the role of priors in guiding adaptive behaviour to the realm of attentive perception, in the absence of specific reward-related choices. Interestingly, our findings emphasize the preservation of the ability to learn and use regularities over the short term, within a task session, suggesting that instability of mood impacts learning-based attentional guidance when longer-term consolidation and integration of memories is required.

This study demonstrates the feasibility of remote, digital methods for capturing high-frequency, concurrent measures of mood and cognition. Such approaches are essential for elucidating mechanisms underlying dynamic, transdiagnostic symptoms such as mood instability, which may contribute vulnerability to, or maintenance of mood episodes and cognitive deficits, as well as providing targets for future interventions (Harrison et al., 2016). Further, we highlight the importance of developing more sensitive tasks to interrogate both global attentional performance and cognitive stability.

## Supplementary Material

Supplementary Material

## Figures and Tables

**Table 1:** Baseline demographics of analysed participants

Table 1. Baseline demographics of analysed participants	High MDQ (*n*=37)	Low MDQ (*n*=37)	*Group differences*
**Age (mean, range)**	25 (18–46)	25 (18–49)	*t*(72)=.22, *p*=.83
**Female sex (n, %)**	24 (65%)	24 (65%)	χ(1)=.00, *p*=1.00
**MDQ score (median, range)**	9 (7–13)	1 (0–4)	*W*=.0, *p*<.001
**Affect Intensity Measure (AIM) (median, range)**	145 (87–200)	140 (55–182)	*W*=430.5.*p*=.02
**Affective Lability Scale (ALS-SF) (median, range)**	41 (18–59)	20 (18–40)	*W*=106.5, *p*<.001
ALS-SF – depression/mania (median, range)	21 (8–30)	10 (8–20)	*W*=136.5, *p*<.001
ALS-SF – anxiety/depression (median, range)	12 (5–18)	5 (5–10)	*W*=123, *p*<.001
ALS-SF – anger (median, range)	8 (5–17)	5 (5–10)	*W*=197, *p*<.001
Tasks completed/50 (median, range)*	46 (31–50)	47 (35–50)	*W*=728, *p=.26*
Lifetime DSM-IV disorders (n, %)**	9 (24.3%)	0 (0%)	-
BD-II	2 (5.4%)	-	-
BD-Not Otherwise Specified (NOS)	2 (5.4%)	-	-
Major Depressive Disorder	6 (16.2%)	-	-
PTSD	1 (2.7%)	-	-
Past alcohol dependence	2 (5.4%)	-	-

* Calculation of compliance only includes data if mood ratings and cognitive task data were both completed on the same day.

** Five participants received 1 diagnosis of a DSM-IV Axis-1 Disorder, and four participants had received 2 diagnoses of DSM-IV Axis-1 Disorders.
