# Supplementary material for: Implicit learning across varying temporal scales in individuals with and without mood instability
Source: J Affect Disord. Author manuscript; Available in PMC 2026 Mar 4. (PMC7618815; doi:10.1016/j.jad.2025.120728)
Supplement: Supplementary Material [file EMS212488-supplement-Supplementary_Material.pdf]

Supplementary material

Methods

Participant exclusion criteria

Exclusion criteria included participants outside criteria for low or high MDQ groups; those currently pregnant or breastfeeding; a history of, or current neurological condition; use of lithium, antidepressants, antipsychotic, or anticonvulsant medication in the past 6 weeks; and current substance abuse. The DSM-IV SCID-I was used to assess all participants for current or prior psychiatric disorder during their in-person baseline visit. In the low MDQ group, participants were excluded if they met criteria for a history of, or current DSM-IV axis I psychiatric disorder or had a first degree relative with BD. In the high MDQ group, participants were excluded if they met criteria for a history of, or current DSM-IV axis I psychiatric disorder other than BD I or II, MDD or anxiety disorder.

Visual attention task

All participants completed one practice block of 18 trials before the task began. On each trial, a white fixation cross was presented on a black screen for 1000 ms. Following fixation, there was an interval of 100 ms before presentation of the stimulus display. Stimuli were presented in six possible fixed spatial locations around an invisible circle (radius = 7.5° of visual angle). Target stimuli could be any letters from a set of 20 capital letters (ABDEFGHJKLMNOPRSTVXZ) chosen randomly on any given trial. Digit distractors could be any number from 1-9 chosen randomly. Immediately following stimulus presentation, a black-and-white mask appeared at all six spatial locations. The mask appeared for 500 ms before participants were prompted to record any letters they had seen by typing them into a standard keyboard in any order. After recording all letters identified in one trial, participants pressed 'enter' to advance to the next trial.

The task was presented on a monitor of 530 mm width and 297 mm height, with a screen resolution of 1920 x 1080 and refresh rate of 100 Hz, allowing display times to vary by 10 ms.

Incremental contextual cueing task

Across conditions, the locations of targets were restricted to ten possible predetermined locations so that targets did not appear at the border, or in the centre of the invisible grid. The

ten target locations were counterbalanced among conditions. There were no limitations for the location of distractors, including target locations used in other configurations. The orientation of targets and distractors was selected randomly from one of four rotations (0, 90, 180 or 270 degrees) and was the same across repetitions of the same configuration.

The task was completed on an iPad 2 with a 9.7-inch (diagonal) screen and 1024 x 768 pixel display. Assuming a 40-cm viewing distance, the visual search array appeared within an invisible 8 x 6 grid of possible locations that subtended approximately 21 x 28 degrees in visual angle at screen centre. Within each invisible grid of the 8 x 6 display, individual stimuli could appear in the centre, or was randomly jittered within +/- 0.6 visual degrees (separately for x and y) from the centre. The jitter was the same for a given configuration when repeated. The size of the stimuli was 4.89mm, 0.7 x 0.7 degrees in visual angle (maximum of 25.8 x 25.8 pixels).

Results

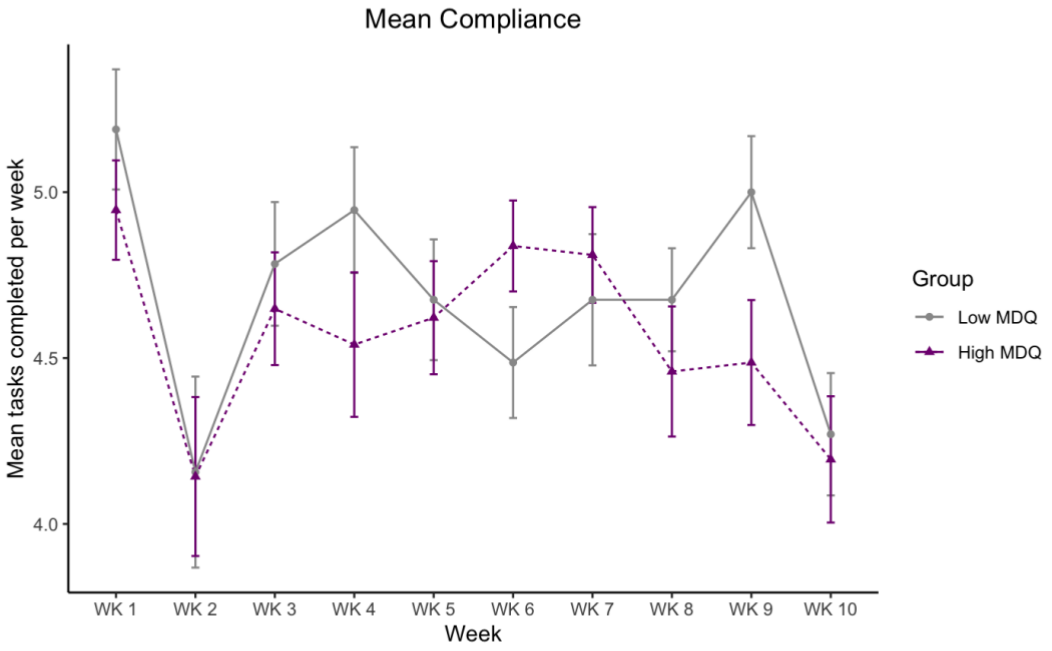

Supplementary Figure 1. Compliance to mood prompts and contextual cueing task. Mean compliance across all weeks of the study, for high and low MDQ groups. Only days in which both mood ratings and task data were submitted are included in calculation of compliance. Error bars represent standard error (SE).

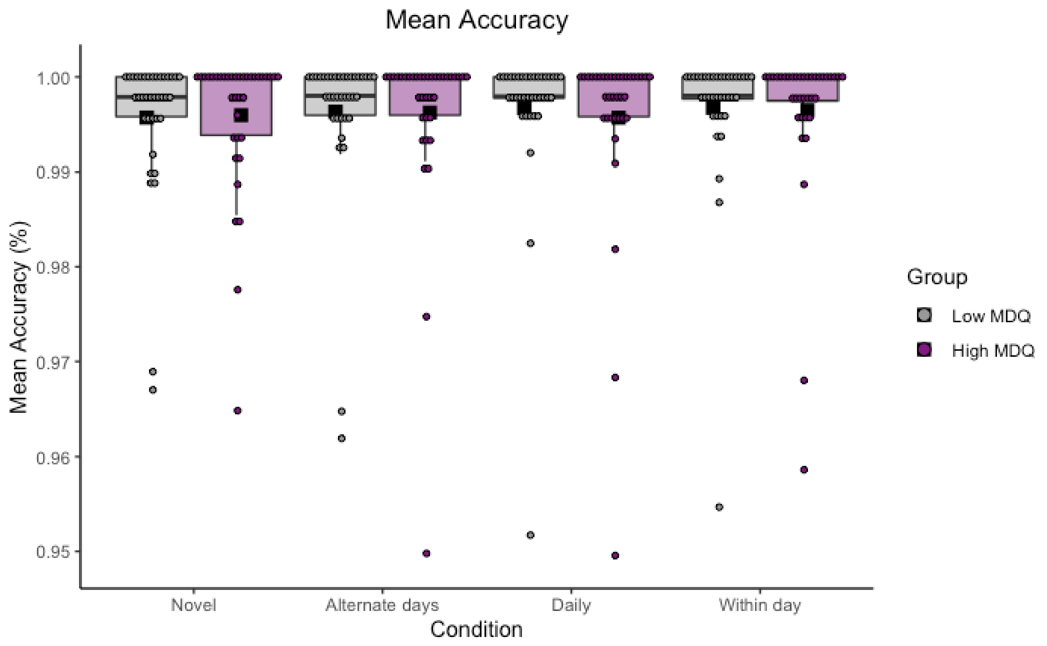

Supplementary Figure 2. Contextual cueing task - accuracy. Accuracy in locating the target 'T', across all trials of the study period, within each condition. Each dot represents the mean accuracy of individuals.

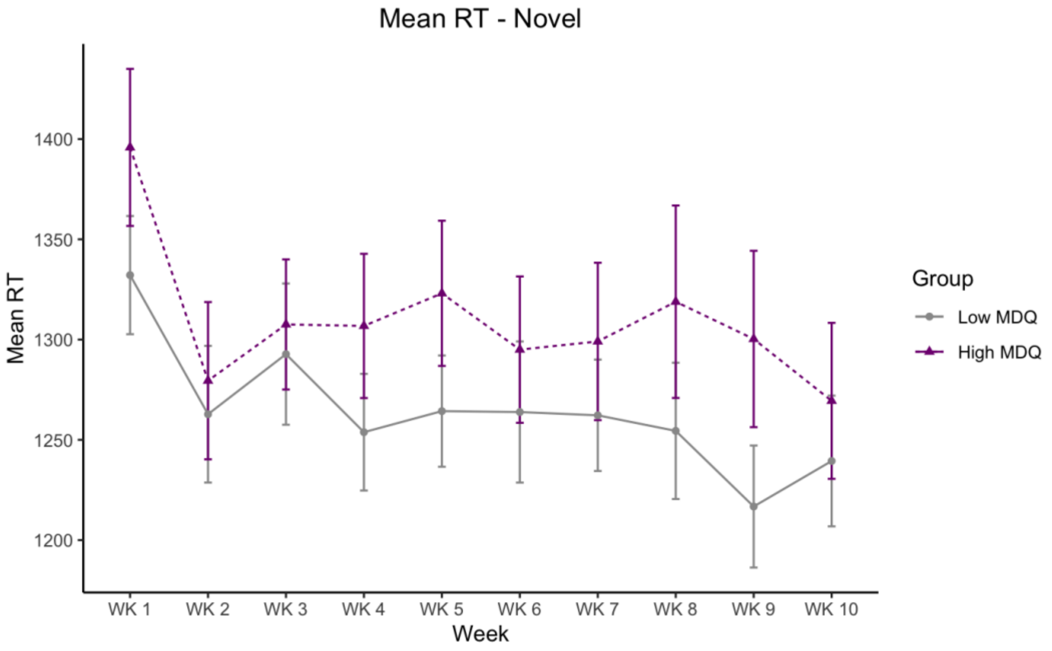

Supplementary Figure 3. **Contextual cueing task - RT to novel trials.** Mean RT on novel trials across the 10 weeks for high and low MDQ groups. Error bars represent standard error (SE).

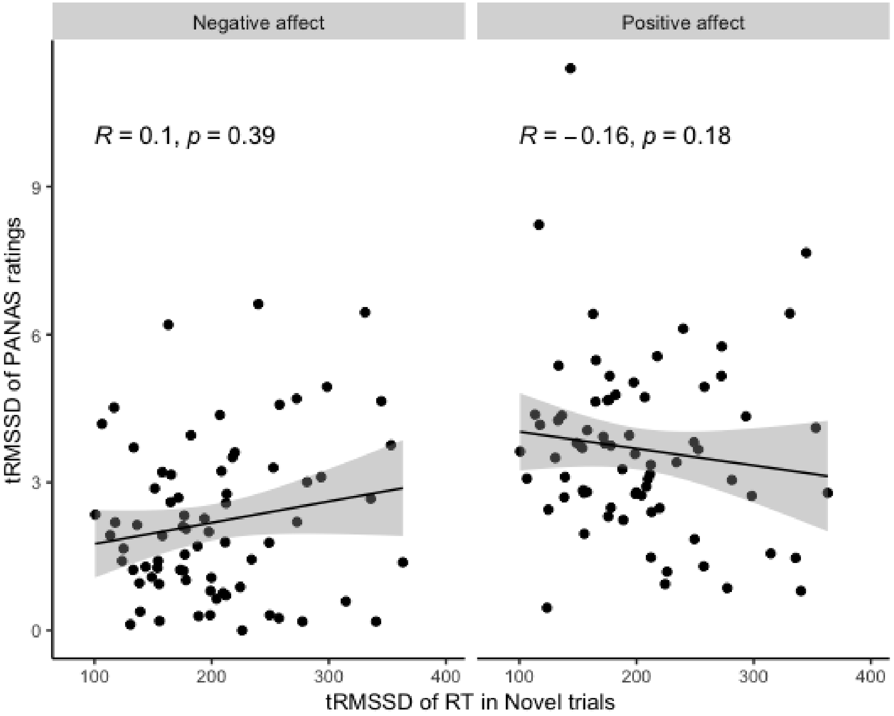

Supplementary Figure 4. Correlation between instability in RT to novel trials and mood. Spearman correlation between overall instability in RTs to identifying novel targets and overall instability in negative and positive mood. Each datapoint represents a participant.

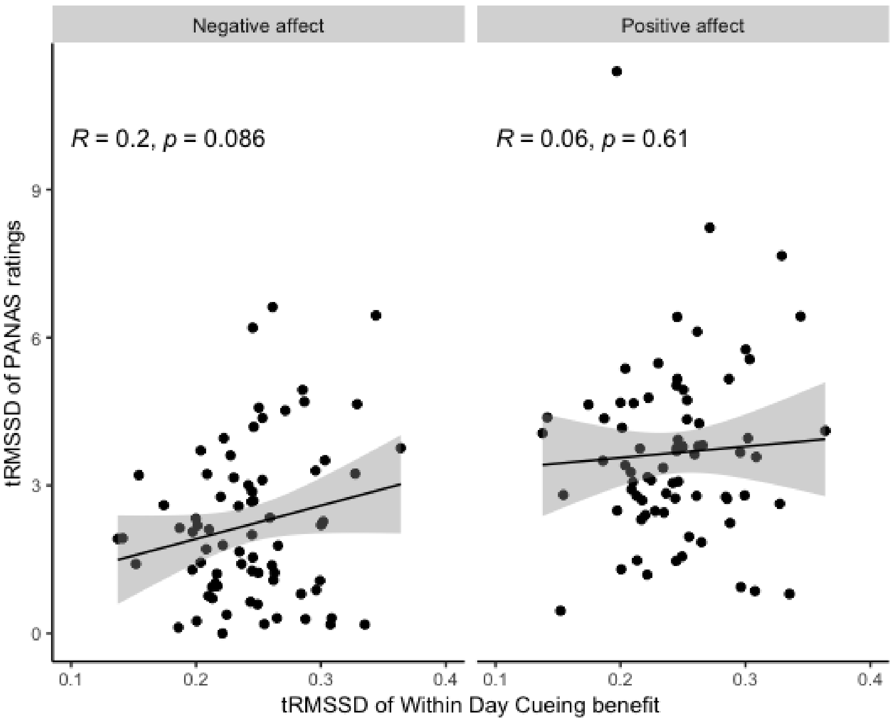

Supplementary Figure 5. Correlation between instability in within day contextual cueing benefit and mood. Pearson correlation between instability in the within day cueing benefit (difference in the slope between within and novel trials each day) and instability in negative and positive mood. Each datapoint represents a participant.

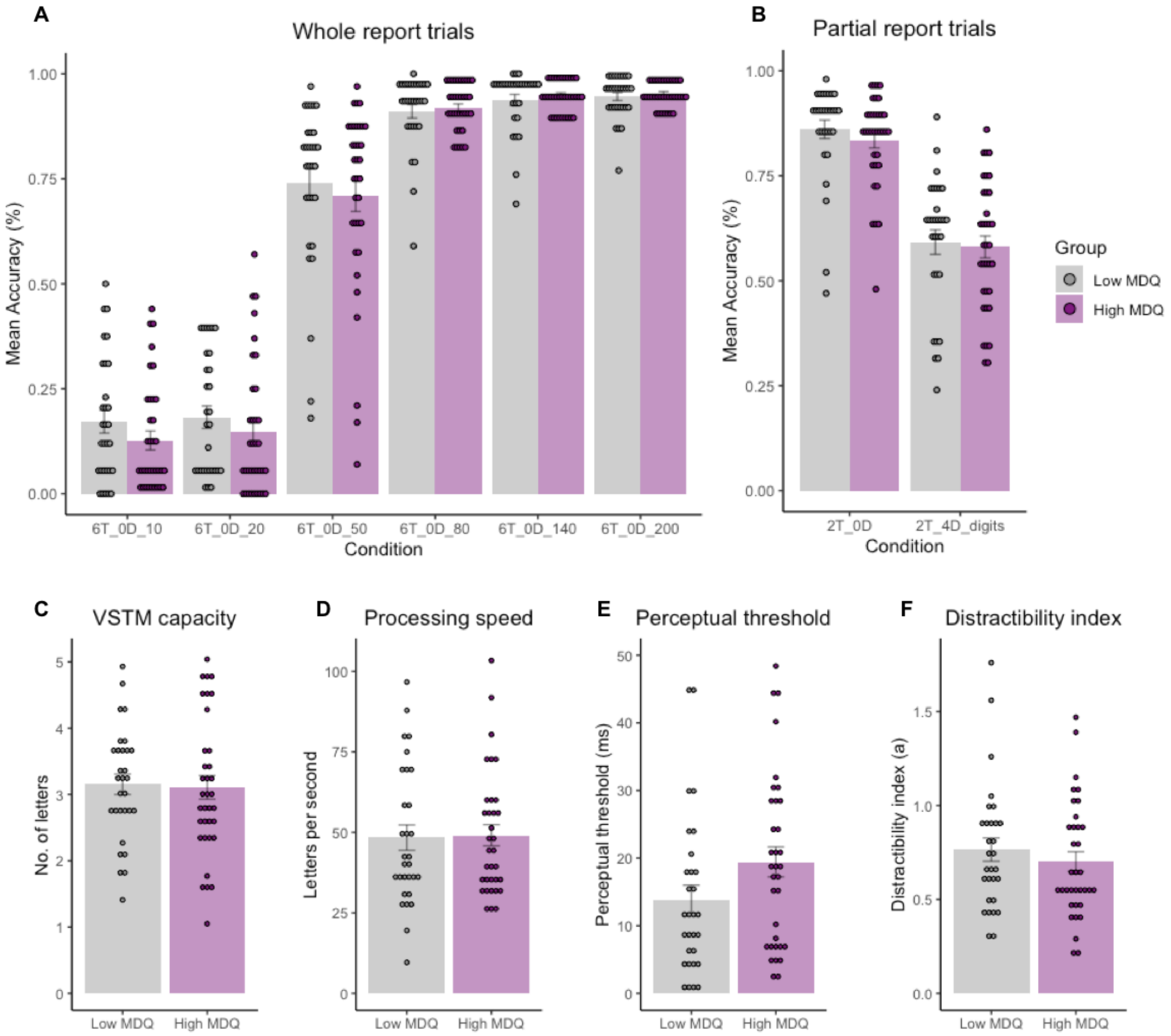

Supplementary Figure 6. **TVA outcomes. A.** Mean accuracy (% of correctly reported target letters) across stimulus exposure durations six-target whole report trials **B.** Mean accuracy in the 2T-0D and partial report distractor condition (2T-4D/digits). **C-F.** Model fits for whole report data including VSTM capacity (*K*), processing speed (*C*), perceptual threshold (*t*0), and distractibility index (α). Values of α close to zero indicate no distractibility/efficient selection of targets and values close to one indicate high distractibility with no prioritisation of targets compared with distractors. Note that one participant (high MDQ) was excluded from *t0* due to abnormally low estimates in perceptual threshold (less than 0 ms) and one participant (high MDQ) was excluded from *C* due to an abnormally high estimate of visual processing speed (>200 letters per second). Error bars represent the SE of the mean. Grey and purple bars represent means of the low and high MDQ groups respectively.

Supplementary Table 1. Baseline demographics of TVA participants.

Demographics of TVA participants	High MDQ (n=35)	Low MDQ (n=30)	*Group differences*
**Age (mean, range)**	25 (18-46)	25 (18-49)	$t(63)=-.07, p=.94$
**Female sex (n, %)**	23 (66%)	24 (80%)	$\chi(1)=1.01, p=.31$
**MDQ score (median, range)**	9 (7-13)	1 (0-4)	$W=.0, p<.001$
**Affect Intensity Measure (AIM) (median, range)**	145 (87-192)	140 (55-182)	$W=386.5, p=.09$
**Affective Lability Scale (ALS-SF) (median, range)**	41 (18-59)	20 (18-40)	$W=100, p<.001$
ALS-SF – depression/mania (median, range)	21 (8-30)	10 (8-20)	$W=107.5, p<.001$
ALS-SF – anxiety/depression (median, range)	12 (5-18)	5 (5-10)	$W=144.5, p<.001$
ALS-SF – anger (median, range)	8 (5-15)	5 (5-10)	$W=174, p<.001$
**tRMSSD negative affect (I-PANAS-SF) (mean, SD)**	2.83 (1.55)	1.47 (1.26)	$t(63)=-3.83, p<.001$
**tRMSSD positive affect (I-PANAS-SF) (mean, SD)**	4.02 (1.51)	3.34 (2.08)	$t(63)=-1.54, p=.13$
**Mean negative affect (I-PANAS-SF)**	7.81 (2.25)	5.84 (1.05)	$t(63)=-4.39, p<.001$
**Mean positive affect (I-PANAS-SF)**	11.95 (2.62)	11.71 (3.68)	$t(63)=-.29, p=.77$
**Lifetime DSM-IV disorders (n, %)****	9 (25.7%)	0 (0%)	-
BD-II	2 (5.7%)	-	-
BD-Not Otherwise Specified (NOS)	2 (5.7%)	-	-
Major Depressive Disorder	6 (17.1%)	-	-
PTSD	1 (2.9%)	-	-
Past alcohol dependence	2 (5.7%)	-	-

** Five participants had received 1 diagnosis of a DSM-IV Axis-1 Disorder, and four participants had received 2 diagnoses of DSM-IV Axis-1 Disorders.

Supplementary Table 2. Outcomes of TVA model parameters

TVA model parameters	High MDQ (*n*=35)	Low MDQ (*n*=30)
**Whole report**		
Visual short term memory capacity, *K* (mean, SD)	3.11 (1.05)	3.15 (.85)
Visual processing speed, *C* (mean, SD)	49.1 (19.32)	48.38 (21.54)
Perceptual threshold, *t*0 (mean, SD)	19.44 (12.88)	13.84 (11.84)
**Partial Report**		
Distractibility index, α (mean, SD)	.70 (.31)	.77 (.34)
